# 

**Published:** 1916-09

**Authors:** 


### ARMY MEDICAL CORPS EXAMINATION.

The Surgeon General of the Army announces that preliminary examinations for appointment of first lieutenants in the Army Medical Corps will be held early in Jan., 1917, at points to be hereafter designated.

Full information concerning this examination can be procured upon application to the "Surgeon General, U. S. Army, Washington, D. C." The essential requirements to secure an invitation are that the applicant shall be a citizen of the United States, between 22 and 32 years of age at time of receiving commission in Medical Corps, a graduate of a medical school legally authorized to confer the degree of Doctor of Medicine, of good moral character and habits, and shall have had at least one year's hospital training as an interne, after graduation. Applicants who are serving this post-graduate interneship and con complete same before Oct. 1, 1917, can take the January examination. The examination will be held simultaneously throughout the country at points where boards can be convened. Due consideration will be given to localities from which applications are received, in order to lessen the traveling expenses of applicants as much as possible.

In order to perfect all necessary arrangements for the examination, applications should be forwarded without delay to the Surgeon General of the Army.

Cases of neglected aural disease might be indefinitely enumerated not only in my practice, but in that of us all. My object, therefore, in presenting this elementary note is for the purpose of soliciting on the part of my colleagues a continuation of our educational programme.—The Therapeutic Gazette.

---

### NEW SUBSCRIBERS

Dr. J. V. Freeman ------ Fla.
Dr. W. W. Rucks ------ Okla.
Dr. C. J. Baumgartner -- Fla.
Dr. W. R. Grover ---- Fla.
Dr. J. P. Holmes ------ Ga.
Dr. T. E. Greene ------- Ala.
Dr. F. W. Haines ------ Va.
Dr. V. L. Wethersby -- W. Va.
Dr. A. A. Landry ------ La.
Dr. H. C. Smith -------- Va.
Dr. Austin Bell -------- Ky.
Dr. A. P. Hunter ------ Fla.
Dr. J. Brown Farrior ---- Fla.
Dr. J. D. Smythe ------ Miss.
Drs. Thraye & Lewis -- Texas
Dr. E. C. Armstrong -- Miss.
Dr. E. E. Smith ------ N. C.
Dr. B. C. Fry -------- La.
Dr. Edward Smoak ------ Fla.
Dr. R. L. Monroe ------ Fla.
Dr. W. W. Battey ------ Ga.
Dr. P. Y. Duckett ----- Ga.
Dr. G. W. Diggs ------ Miss.
Dr. Wm. Pumpelly ---- Ga.
Dr. A. G. Kern -------- Tenn.
Dr. Lawrence Lee ------ Ga.
Dr. Henry Leidheimer ---- La.
Dr. T. E. Williams ---- La.
Dr. J. C. McDaniels ---- Ala.
Dr. A. H. Johnson ------ La.
Drs. O'Donnell & Gray -- La.
Dr. L. A. Baker -------- Ga.
Dr. H. E. Cunningham -- Ala.
Dr. J. N. Furnis------- Ala.
Dr. P. H. White -------- Ala.
Dr. M. C. Beverett ------ Fla.
Dr. Jno. F. Chamberlian-Miss.
Dr. Herbert Wiggins ---- Ala.
Dr. Pearse O'Leary -- Miss.
Dr. Geo. L. Echols ------ Ga.
Dr. G. M. Huckaby ------ Ala.
Dr. E. C. Thompson ---- Ala.
Dr. F. L. Summers ---- Miss.
Dr. G. M. Huckaby ---- Ala.
Dr. A. D. Simington ---- Ala.
Dr. G. M. Taylor ------ Ala.
Dr. E. B. Leddle ------ Miss.
Dr. M. S. Davis -------- Ala.
Dr. T. B. Lewis ------ Miss.
Dr. Wm. K. Gray ------ Miss.
Dr. W. G. Page -------- Ala.
Dr. J. L. McCormack -- Miss.
Dr. J. E. Walker ---- Miss.
Dr. M. D. Smith -------- Ala.
Dr. F. M. Hill -------- Ga.
Dr. Richard Binnion ---- Ga.
Dr. J. H. Whiteside ---- Ga.
Dr. J. A. Thrash ------ Ga.
Drs. Sparkman & Sparkman ------------------ Miss.
Dr. I. W. Brown -------- Miss.
Dr. J. W. Cox -------- Miss.
Dr. J. V. James --------- Miss.
Dr. W. L. Kennedy --- Miss.
Dr. J. T. Bailey ------ Miss.
Dr. J. C. Crawley ---- Miss.
Dr. C. M. Franklin ---- Ala.
Dr. F. W. Samuels ----- Ky.
Dr. C. B. Crawford ------ Ga.
Dr. W. P. Hughes ------ Ky.
Dr. E. R. Palmer ------ Ky.
Dr. R. L. Miller -------- Ga.
Dr. M. S. Davis -------- Ky.
Dr. E. E. Underwood ---- Ky.
Dr. W. H. Hadaway ------ Ga.
Dr. W. B. Jordan ------- Ga.
Dr. W. C. Howell ------- Ala.
Dr. E. Iseman ---------- Ga.

